# Should Scotland provide genome-wide sequencing for the diagnosis of rare developmental disorders? A cost-effectiveness analysis

**DOI:** 10.1007/s10198-024-01717-8

**Published:** 2024-09-09

**Authors:** Michael Abbott, Mandy Ryan, Rodolfo Hernández, Lynda McKenzie, Sebastian Heidenreich, Lynne Hocking, Caroline Clark, Morad Ansari, David Moore, Anne Lampe, Ruth McGowan, Jonathan Berg, Zosia Miedzybrodzka

**Affiliations:** 1 Health Economics Research Unit, University of Aberdeen, Aberdeen, UK; 2 Evidera Inc., London, UK; 3 Department of Medical Genetics, University of Aberdeen, Aberdeen, UK; 4 NHS Grampian Regional Genetics Service, Aberdeen Royal Infirmary, Aberdeen, UK; 5 South East Scotland Genetic Service, NHS Lothian, Edinburgh, UK; 6 West of Scotland Centre for Genomic Medicine, QEUH, Glasgow, UK; 7 NHS Tayside Genetics Service, Dundee, UK

**Keywords:** Economic evaluation, Cost effectiveness, Genetics, Genomics, Rare conditions

## Abstract

**Aims:**

This study aims to evaluate the cost effectiveness of genetic and genomic testing strategies for the diagnosis of rare developmental disorders in NHS Scotland.

**Methods:**

Six genetic and genomic testing strategies were evaluated using a decision tree model. First-line, second-line and last-resort trio genome sequencing (GS), and second-line and last-resort trio exome sequencing (ES) were compared with standard genetic testing. The cost effectiveness of each strategy was expressed in terms of incremental cost per additional diagnosis. The impact of uncertainty on cost-effectiveness results was explored using deterministic and probabilistic sensitivity analysis.

**Results:**

2nd-line ES was a cost-saving option, increasing diagnostic yield by 13.9% and decreasing cost by £1027 per trio compared to standard genetic testing. Compared to ES, strategies involving GS increased costs significantly, with only a moderate or zero improvement in diagnostic yield. Sensitivity analysis indicated that significant reductions in cost or improvements in diagnostic yield are required before 1st-line GS becomes cost effective.

**Conclusion:**

2nd-line ES (after chromosomal microarray; replacing gene panel testing) for the diagnosis of developmental disorders is a cost-saving option for the Scottish NHS. Ongoing economic evaluation is required to monitor the evolving cost and diagnostic yield of GS and ES over time.

**Supplementary Information:**

The online version contains supplementary material available at 10.1007/s10198-024-01717-8.

## Key Points

- This study evaluated the cost effectiveness of six testing strategies for the diagnosis of rare developmental disorders in Scotland.
- It was found that 2nd-line exome sequencing was less costly and more effective than standard genetic testing.
- Genome sequencing marginally increased diagnostic yield compared to exome sequencing, but increased costs significantly.
- NHS Scotland should continue to invest in exome sequencing for the diagnosis of rare genetic conditions, and should monitor the evolving cost and diagnostic yield of genome sequencing over time.

## Introduction

Approximately 2–5% of children are born with rare developmental disorders, or manifest symptoms during childhood [1]. Although developmental disorders often have heterogeneous clinical presentations, the most commonly observed features consist of intellectual disability/developmental delay and/or congenital malformations [2]. Many developmental disorders are life-threatening and multi-system, with a profound impact on the quality of life and well-being of patients and families. The combination of the diversity of developmental disorders and the clinical expertise required to diagnose them creates a challenge for publicly funded healthcare systems with increasingly strained budgets. Given that a significant proportion of developmental disorders are believed to have a genetic cause [3, 4], increasing access to genetic diagnostic testing is a key policy objective of the Scottish Government [5].

Many patients and families with undiagnosed developmental disorders undergo a long, stressful, and costly series of clinical investigations and genetic testing in search of a diagnosis. First-line genetic testing typically involves chromosomal microarray (CMA), and Fragile X testing. When clinical investigations and first-line genetic testing fail to reach a diagnosis, individuals undergo an iterative series of targeted gene panels based on clinical phenotype. Gene panel testing can be a useful diagnostic tool for genetically heterogeneous conditions, or when an individual has clinical features which may fit more than one condition. However, the chance of obtaining a diagnosis from gene panel testing depends on: (i) the clinician requesting the correct gene panel based on the patient’s phenotype; and (ii) the gene causing the rare condition being on the existing gene panel. As a result, the diagnostic yield (proportion of cases receiving a genetic diagnosis) of gene panels may not be optimal [6]. The iterative series of clinical and genetic testing has been labelled the ‘diagnostic odyssey.’ This refers to the time taken between the first presentation at health care services and receiving a correct medical diagnosis [2]. Historically, Scottish patients have waited an average of four years to receive a genetic diagnosis for their rare condition, with many never receiving one [7].

Advancements in genomics offer promising opportunities to end or shorten the diagnostic odyssey for the diagnosis of rare developmental disorders. The comprehensive sequence analysis of a person’s entire genome (genome sequencing – GS), or the protein-coding region of a person’s genome (exome sequencing – ES), may enable quicker diagnosis for rare conditions due to its higher diagnostic yield than standard gene panels [8, 9]. It is well known that over 2,000 genes are associated with developmental disorders [9]. GS and ES offer an opportunity to analyse these genes efficiently. In Scotland, both GS and ES have been offered in a research context. Firstly, 1250 children with undiagnosed developmental disorders were offered ES via the Deciphering Developmental Disorders (DDD) study [10]. A further 385 patients and families were offered panel-based analysis of GS via the Scottish Genomes Partnership’s (SGP) involvement in the UK 100,000 Genomes Project [11, 12]. At present, analysis of the Developmental Disorder Genotype-to-Phenotype (DDG2P) gene panel [13] from exome sequence data is currently offered in NHS Scotland as a specialised diagnostic service to individuals presenting with a severe developmental disorder.

The economic evidence on the value for money offered by genome-wide sequencing for rare disease diagnosis is growing [14]. Despite this growing literature, the cost effectiveness of genome-wide sequencing remains highly uncertain, varying significantly depending on the study context, patient population and strategies evaluated. The health economic evidence in a Scottish context is particularly limited, with only one economic evaluation in the United Kingdom (UK) [15], and none in Scotland. Given that Scotland operates under a devolved health care budget, separate from other parts of the UK, health economic evidence is required to inform the development of a Scottish genomic testing strategy.

Abbott et al. [7] present preliminary estimates of the cost of GS versus the standard genetic testing pathway for rare disease diagnosis in Scotland. Trio GS (where *trio* refers to sequencing DNA samples from a child plus two biological parents) was estimated to cost £6625, compared to £1841 per patient for standard genetic testing. However, the study did not evaluate the cost of alternative genome-wide sequencing options, including trio ES. Additionally, the study stopped short of conducting an incremental cost-effectiveness analysis of alternative strategies in terms of their diagnostic yield. Building on these preliminary findings, this study aims to assess the cost effectiveness of genetic and genomic testing strategies for the diagnosis of rare developmental disorders in Scotland.

## Materials and Methods

**Fig. 1:**
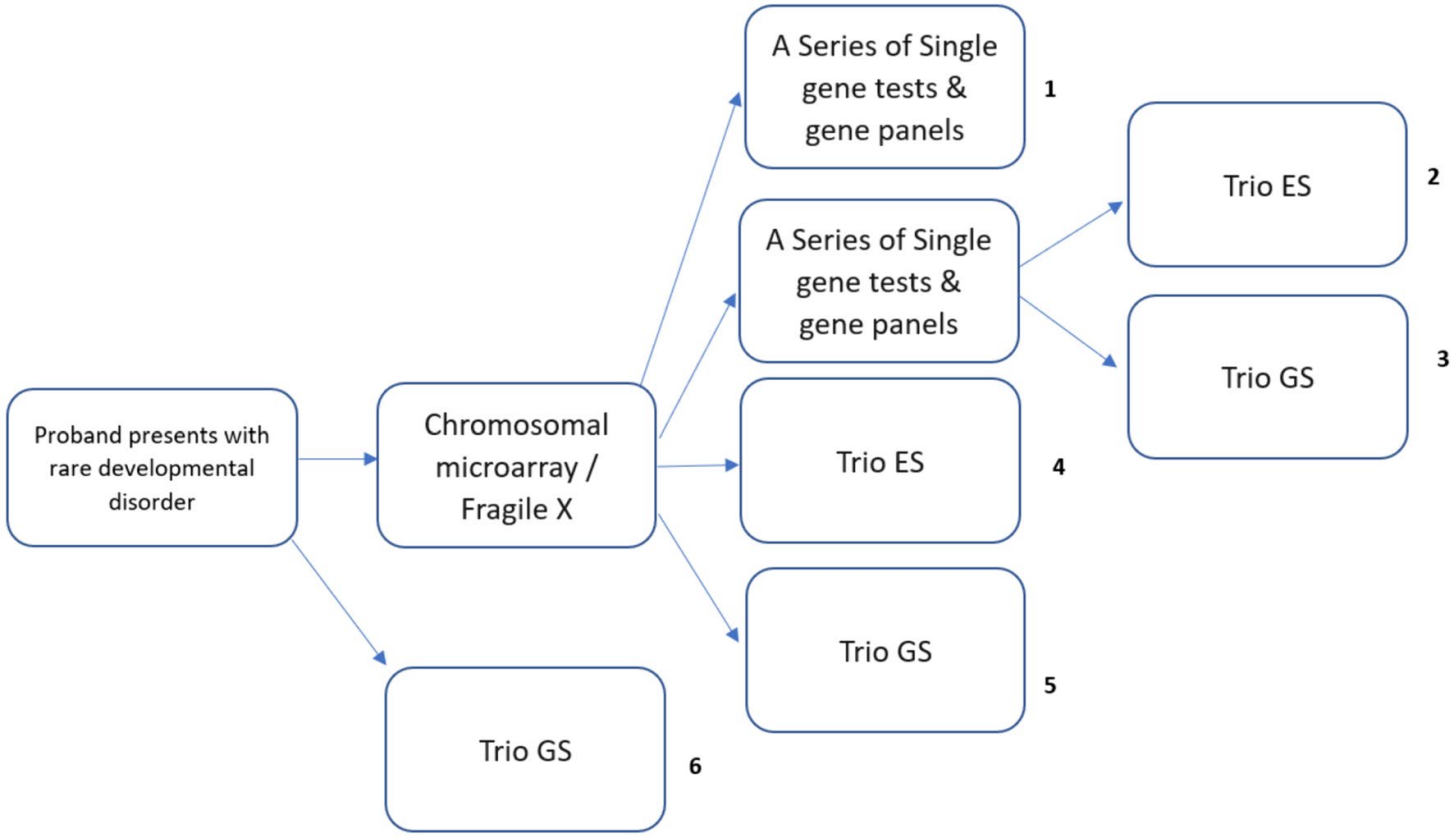
Simplified schematic of cost-effectiveness analysis model structure

A decision tree model was developed using TreeAge Pro (TreeAge Pro ® 2021). Six genetic and genomic testing strategies were evaluated for the diagnosis of rare developmental disorders, from a Scottish health care system perspective. Alternative configurations of standard genetic testing, GS and ES were evaluated at varying time points in the diagnostic pathway. The strategies were selected based on plausible alternatives which could be delivered in Scottish clinical practice and were informed and validated by expert clinical opinion. A simplified schematic of the model structure is presented in Fig. 1.

*Standard genetic testing* refers to the historical ‘diagnostic odyssey’ of chromosomal microarray, Fragile X, single gene, and gene panel testing. Although this strategy was labelled *standard genetic testing*, it is not currently ‘standard care’ in NHS Scotland, given that a trio ES service is now offered for severe developmental delay. Standard genetic testing was included in the model as a means of estimating the historical cost and diagnostic yield of the diagnostic odyssey and was assumed to involve singleton (proband-only, without samples from parents or relatives) testing.

In addition to standard testing, trio GS was evaluated as a last-resort test (after all standard testing fails to reach a diagnosis), as a 2nd-line test (replacing single gene and gene panel testing) and as a 1st-line test (replacing all standard genetic testing). Trio ES was evaluated as a last-resort test and 2nd-line test. 1st-line trio ES was not evaluated because, at the time of model conception and development, expert clinical opinion indicated that chromosomal microarray testing would always occur prior to trio ES. Despite recent developments indicating that trio ES may be able to replace array testing [16–18], there was insufficient data on the cost and diagnostic yield of first-line ES to reliably model this strategy. In all strategies, ES and GS were assumed to involve trio testing, using DNA samples from the proband plus two biological parents. This reflected the diagnosis of rare developmental disorders in Scotland, where the majority of cases involve trio testing.

NHS Scotland data from the SGP and DDD research studies and the NHS Scotland DDG2P pipeline, as well as review of the clinical literature, informed the model. Whilst the SGP and DDD studies recruited a broad range of rare disease phenotypes, we focus on rare developmental disorders due to the significantly larger sample size for developmental disorders compared to other rare conditions. This increased the precision of cost, diagnostic yield, and cost-effectiveness estimates.

Table 1 summarises the cost and diagnostic yield parameters used in the model. Further information on all model inputs, assumptions and distributions is available in the *Online Supplementary Material.* In Table 1, all cost and diagnostic yield estimates apply to individual tests rather than entire testing strategies. For example, the diagnostic yield of last-resort trio GS does not include the yield of prior chromosomal microarray, Fragile X and gene panel testing.

**Table 1:** Summary of cost effectiveness analysis model inputs

Genetic/Genomic test	Cost* (95% CI)	Diagnostic yield (95% CI)
1st-line chromosomal microarray and Fragile X testing	£386 (£358–£414)	0.10 (0.09–0.12)
2nd-line single gene tests and gene panels	£2275 (£1836–£2948)	0.21 (0.14–0.29)
Trio genome sequencing	**Scottish Genomes Partnership Pipeline: £5576 (£5018–£6133) ** Alternative Pipeline**: £3781 (£3403–£4159)	**1st-line:** 0.46 (0.36–0.57) **2nd-line:** 0.40 (0.33–0.47) **Last-resort:** 0.23 (0.14–0.32)
Trio exome sequencing	£1153 (£1060–£1245)	**2nd-line:** 0.37 (0.27–0.49) **Last-resort:** 0.21 (0.13–0.29)

*All costs are reported in 2022 British Pounds Sterling and include VAT

**Outsourcing sequencing, bioinformatic analysis and data storage to Genomics England

The words in bold indicate alternative ways in which trio genome sequencing could be delivered

### Costing standard genetic testing

The cost of standard genetic testing was estimated by attaching unit costs to the pre-genomic testing histories of SGP and DDD study participants. Testing histories included chromosomal microarray, Fragile X, single gene tests and gene panels. Genetic testing costs were derived from a workload unit-based method developed by the UK Genetic Testing Network [19], with each genetic test placed into one of eight costing bands reflecting the complexity of the test in terms of staff time, laboratory inputs and overheads. The cost of clinic visits depended on whether the appointment was with a genetics consultant (£396 per appointment [20]) or counsellor/nurse, where the Personal Social Services Research Unit (PSSRU) cost per minute [21] was used.

### Costing trio genome sequencing and exome sequencing

The workload unit costing method has not been updated to include GS and ES. Trio GS and ES costs were estimated using a combination of micro-costing at Scotland’s four regional genetics centres and charges to the regional centres from third-party providers. Although charges are not necessarily indicative of costs [22], the charges from third-party providers reflect the price which NHS Scotland would need to pay to deliver a genome-wide sequencing pipeline, including sequencing and bioinformatic analysis. These charges therefore reflect the opportunity cost of NHS Scotland’s expenditure and the health care system perspective of the analysis. The base-case analysis used trio GS costs based on the SGP study GS pipeline, updated for both inflation and changes in the cost of delivering GS in clinical practice rather than in a research context. The cost of trio ES was estimated using micro-costing at South East Scotland Genetic Service, where trio ES is currently offered for severe developmental disorders.

### Diagnostic yield

Diagnostic yield (the proportion of cases receiving a genetic diagnosis) was used as the clinical effectiveness measure. A combination of primary data from the SGP and DDD studies and the DDG2P pipeline, as well as systematic review data and expert clinical opinion, was used to estimate the diagnostic yield of each strategy. For standard genetic testing, diagnostic yield estimates were obtained from systematic review data [8] and expert clinical opinion. The SGP and DDD research studies provided estimates of the diagnostic yield of trio GS and ES *as a last-resort test,* where the eligibility criteria typically required exhaustive standard genetic testing with residual unmet diagnostic need. The NHS Scotland DDG2P trio ES pipeline provided insight into the diagnostic yield of trio ES *as a 2nd-line test*, after chromosomal microarray and/or Fragile X testing had failed to reach a diagnosis. No primary data was available for the diagnostic yield of 1st-line and 2nd-line GS in NHS Scotland; estimates for these strategies were obtained from systematic review data [8].

### Cost-effectiveness analysis

The incremental cost per additional diagnosis (ICAD) was estimated as:

$$ICAD = \frac{{C_{1} - C_{0} }}{{DY_{1} - DY_{0} }}$$

where C_1_ and DY_1_ are the mean costs and diagnostic yield for a given testing strategy, and C_0_ and DY_0_ are the mean costs and diagnostic yield of the next-best alternative strategy. Cost-effectiveness results were plotted on an efficiency frontier, connecting strategies that are successively more costly and more effective [23, 24]. Strategies on the efficiency frontier may be considered cost effective, depending on the decision maker’s willingness to pay (WTP) per additional diagnosis. Strategies which are not on the efficiency frontier are ‘inefficient’ or ‘dominated,’ in that an alternative strategy could be implemented with a lower cost and higher effectiveness.

Expert clinical opinion indicated that the six genetic and genomic testing strategies could be delivered within three years in clinical practice. The cost effectiveness of each strategy was thus evaluated over a three-year time horizon, starting at the point of referral to the clinical genetics service, and ending after all testing has/has not reached a diagnosis. Following the recommendation of the National Institute for Health and Care Excellence (NICE), all cost and diagnostic yield parameters were discounted at an annual rate of 3.5% [25].

### Modelling uncertainty: sensitivity analysis

In the base-case analysis, trio GS costs were based on an updated SGP study pipeline, with a cost of £5576 per trio. One-way (deterministic) sensitivity analysis explored the impact of a second GS costing option, involving outsourcing sequencing, bioinformatic analysis and data storage to Genomics England, with a cost of £3781 per trio. A threshold analysis investigated how much each cost or diagnostic yield input would need to increase or decrease before the optimal strategy changes. This analysis requires knowledge of the decision maker’s *willingness to pay* (WTP) per additional diagnosis. Drawing on methodology used to estimate the value of a statistical life [26], we estimated an implied WTP based on historical genetic testing policy in Scotland. Dividing the average standard genetic testing cost (£2429) by its diagnostic yield (28.2%) implies that decision-makers have (historically) been willing to pay £8613 per diagnosis.

Probabilistic sensitivity analysis (PSA), with a Cost-Effectiveness Acceptability Curve (CEAC) generated for each testing strategy, estimated the probability that each strategy is cost effective for a range of WTP values per additional diagnosis, from £0 to £100,000. In addition to addressing uncertainty in the cost and diagnostic yield of each strategy, this analysis also addresses uncertainty in the decision maker’s WTP per additional diagnosis.

## Results

Base-case cost effectiveness results are presented in Table 2*,* with strategies reported in ascending cost order. Costs and diagnostic yields are reported for the *entire strategy* (rather than for individual tests). For example, for 2nd-line ES, the cost (£1402) and diagnostic yield (42.1%) include the expected cost and diagnostic yield of prior chromosomal microarray and/or Fragile X testing. The cost and diagnostic yield of each strategy are also discounted at a rate of 3.5% per annum after year 1. As a result, future costs and diagnoses are weighted less than current costs and diagnoses.

**Table 2:** Base case cost-effectiveness results: GS cost = £5576 per trio

Strategy	Cost^a^	Diagnostic yield^a^	Incremental cost	Incremental yield	Incremental cost per additional diagnosis (ICAD)^b^
2nd-Line ES	£1402	42.1%	–	–	–
Standard genetic testing	£2429	28.2%	£1027	– 13.9%	Dominated by 2nd-line ES
Last-resort ES	£3168	44.8%	£1766	2.7%	£65,407^**c**^
2nd-Line GS	£5194	44.7%	£2,026	– 0.1%	Dominated by last-resort ES
1st-Line GS	£5576	46.0%	£2,408	1.2%	£200,666^**d**^
Last-Resort GS	£6112	46.1%	£536	0.1%	£536,000^**e**^

^a^Cost and diagnostic yield discounted at 3.5% per annum after year 1

^b^*ICAD =* Change in cost/change in diagnostic yield

^c^£65,407 = (£3168–£1,402) / (44.8–42.1%)

^d^£200,666 = (£5576–£3168) / (46.0–44.8%)

^e^£536,000 = (£6112–£5576) / (46.1–46.0%)

2nd-line ES had the lowest cost, at £1402 per trio, with a diagnostic yield of 42.1%. Standard genetic testing was dominated by 2nd-line ES, with a higher cost (£2429) and lower diagnostic yield (28.2%). Compared to 2nd-line ES, last-resort ES had an incremental cost of £1766, with an incremental diagnostic yield of 2.7%, resulting in an incremental cost per additional diagnosis of £65,407. 2nd-line GS was dominated by last-resort ES, with a higher cost per trio and marginally lower diagnostic yield. First-line GS offered an additional 1.2% diagnostic yield compared to last-resort ES, at an additional cost of £2,408 per trio, resulting in an incremental cost of £200,666 per additional diagnosis. Last resort WGS was the most expensive testing strategy, at £6112 per trio. Compared to first-line GS, last-resort GS had an incremental cost of £536, with an additional yield of 0.1%. This gave an incremental cost per additional diagnosis of £536,000.

**Fig. 2:**
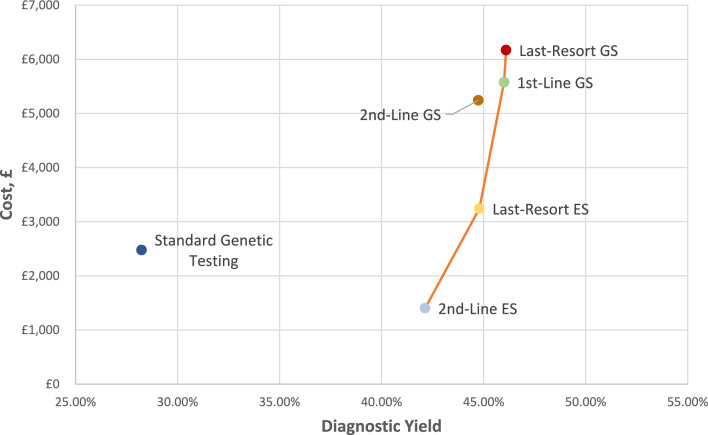
Base case efficiency frontier: GS cost = £5576 per trio

Figure 2 illustrates the expected cost and diagnostic yield of each testing strategy on an efficiency frontier. Strategies which are undominated (2nd-line ES; last-resort ES; 1st-line GS, and last-resort GS) are connected by the orange line, forming the efficiency frontier. These strategies could be considered cost effective, depending on the decision maker’s WTP per additional diagnosis. Strategies which are not on the orange line (standard testing and 2nd-line GS) are ‘inefficient’ or ‘dominated’ strategies, as an alternative strategy could be implemented with a lower cost and higher diagnostic yield.

### Sensitivity analysis: lower GS costs

The base case analysis assumed that trio GS is delivered using an updated version of the trio GS pipeline from the SGP study. To assess the impact of lower GS costs, a second analysis was conducted using a GS cost of £3781 per trio. Table 3 presents the cost-effectiveness results for this lower GS cost.

**Table 3:** Cost-effectiveness results for GS cost = £3781 per trio

Strategy	Cost	Diagnostic yield	Incremental cost	Incremental yield	Incremental cost per additional diagnosis^a^
2nd-Line ES	£1402	42.1%	–	–	–
Standard Genetic Testing	£2429	28.2%	£1027	– 13.9%	Dominated by 2nd-line ES
Last-Resort ES	£3168	44.8%	£1766	2.7%	Dominated by 1st-line GS
2nd-Line GS	£3657	44.7%	£2255	– 0.1%	Dominated by last-resort ES
1st-Line GS	£3781	46.0%	£2379	3.9%	£61,000^**b**^
Last-Resort GS	£4933	46.1%	£1152	0.1%	£1,152,000^**c**^

^a^*ICAD* = Change in cost/change in diagnostic yield

^b^£61,000 = (£3651–£1402) / (46.0–42.1%)

^c^£1,152,000 = (£4933–£3781) / (46.1–46.0%)

2nd-line ES remained a cost-saving option compared to standard genetic testing with a cost saving of £7388 per additional diagnosis (£1027/ – 13.9%). Compared to 2nd-line ES, 1st-line GS increased costs by £2379 per trio, and increased yield by 3.9%. This gave an incremental cost per additional diagnosis of £61,000 for 1st-line GS, compared to 2nd-line ES. Last-resort GS had an incremental cost of £1152 compared to 1st-line GS, with an incremental diagnostic yield of 0.1%. This gave an incremental cost of £1,152,000 per additional diagnosis for last-resort GS.

### Threshold analysis

Assuming a WTP of £8613 per additional diagnosis, it was estimated that, everything else equal:

- The cost of trio GS would need to fall to £1753 per trio before first-line GS becomes cost effective.
- The cost of single gene tests/panels (including clinic visits) would need to fall to £893 before last-resort ES becomes cost effective.
- The yield of first-line GS would need to increase to 89% before 1st-line GS becomes cost effective.
- The yield of second-line GS would need to increase to 87% before second-line GS becomes cost effective.
- Last-resort GS is never a cost-effective option.
- The yield of single gene tests and panels would need to increase to 45% before last-resort ES becomes cost effective.

## Probabilistic sensitivity analysis

The probabilistic sensitivity analysis indicated that, when the base case GS cost of £5576 per trio was used, 2nd-line ES was most likely to be cost effective at WTP values up to £83,000 per additional diagnosis. At the implied WTP value of £8613 per additional diagnosis, 2nd-line ES had a 93% chance of being cost effective. When the lower GS cost of £3781 per trio was used, 2nd-line ES was most likely to be cost effective at WTP values up to £48,000 per additional diagnosis. At the implied WTP value of £8,613 per additional diagnosis, 2nd-line ES had a 73% probability of being cost effective. The cost-effectiveness acceptability curve (CEAC) for each strategy is presented in the *Online Supplementary Material.*

## Discussion

### Informing a Scottish genomic testing strategy

Compared to standard genetic testing, 2nd-line ES (after 1st-line CMA and Fragile X) resulted in a 13.9% increase in diagnostic yield (from 28.2% to 42.1%) and a £1027 decrease in cost (from £2429 to £1402). Strategies involving GS had significant incremental costs, with minimal improvement in diagnostic yield compared to ES. As a result, the incremental cost per additional diagnosis for first-line GS ranged from £61,000 to £200,666, depending on the GS cost. 2nd-line and last-resort GS were not cost-effective testing strategies compared to first-line GS. The evolving cost and diagnostic yield of GS and ES should be carefully monitored within future health economic analyses.

Recent developments, including a press release from Illumina [27], indicate that its NovaSeq X series may reduce the cost of GS significantly. However, it remains unclear how these reductions in GS costs would translate into service delivery in clinical practice. Given this uncertainty, our threshold analysis is pertinent, highlighting that significant reductions in cost and/or improvements in diagnostic yield are required before 1st-line GS becomes a cost-effective testing strategy. Assuming a WTP of £8613 per additional diagnosis, 1st-line GS would need to cost £1753 per trio or have a yield of 89% before it becomes cost effective. The probabilistic sensitivity analysis indicates that, unless the decision-maker’s WTP per additional diagnosis exceeds £83,000 (or £48,000 for the lower GS cost), 2nd-line ES is most likely to be cost effective for the diagnosis of rare developmental disorders.

Comparing our results with other cost-effectiveness analyses of GS for the diagnosis of rare genetic conditions highlights the uncertainty which remains in the value for money offered by genome-wide sequencing. Although our results for trio ES are broadly in line with other studies, finding that early initiation of ES offers substantial cost savings relative to standard genetic testing [28–33], the health economic literature has not reached a consensus regarding the cost effectiveness of GS *relative to ES*. While some studies found that GS ranged from cost-neutral to cost-saving [34–36], others indicated that GS has substantial incremental costs with only modest improvements in diagnostic yield [28, 37].

The heterogeneity in the economic evaluation literature likely reflects the structural, methodological and contextual heterogeneity across studies. The cost effectiveness of GS, ES and standard genetic testing is often estimated using disparate methodological frameworks (cost-effectiveness, cost-utility or cost–benefit analysis), applied to various contexts and patient populations (specific rare conditions, all rare conditions, developmental delay), with inconsistent baseline comparators (comparison with the next-best alternative, standard testing or no testing). This makes it challenging to draw broad comparisons between our results and the economic evaluation literature as a whole. However, it highlights the importance of noting the specific context to which our results apply; the diagnosis of rare developmental disorders in NHS Scotland. We find that, within this context, trio ES offers substantial cost savings relative to standard testing, while trio GS has significant incremental costs and minimal improvement in diagnostic yield relative to ES.

### Limitations

This study evaluated six alternative genetic and genomic testing strategies. These strategies reflected plausible alternatives which could be delivered in Scottish clinical practice and were informed and validated using expert clinical genetics opinion. However, the strategies evaluated may not be exhaustive. For example, trio ES was not evaluated as a first-line test. Recent developments in the ability of trio ES to detect copy-number variations (CNVs) may reduce the need for first-line array testing, making first-line ES a plausible alternative [16–18]. Despite this development, the data on the cost and diagnostic yield of first-line ES was insufficient to model it as a comparator. Additionally, a significant proportion of patients remain undiagnosed after trio ES. It is currently unclear whether these patients could receive trio GS following non-diagnostic ES.

Marshall et al. (2017) described “multiple cascading uncertainties” associated with the economic evaluation of genome-wide sequencing technologies [38]. Among these uncertainties is the evolving cost of GS and ES. We estimated that, using a similar pipeline to the SGP research study, trio GS would cost between £3,781 and £5,576 depending on the sequencing provider. In addition to reductions in the cost of sequencing, improvements have been made in several key areas of GS which may reduce costs compared to the SGP research study. These include reductions in data storage costs, more efficient bioinformatics pipelines and reduced clinical scientist time required for variant interpretation and analysis. As a result, it is unclear whether the trio GS costs estimated in this study reflect current GS costs in a clinical context. In particular, the base-case trio GS costs used in this study were based on early experience of GS in a research study context. However, several of the potential GS cost reductions may also apply to trio ES. Improvements in diagnostic yield would also change the cost-effectiveness results. The diagnostic yield of both GS and ES are likely to improve as we learn more about the structure and function of the human genome.

This study used diagnostic yield as a measure of ‘effectiveness’ of alternative testing strategies. Diagnostic yield was chosen as, within the National Services Division (NSD) of NHS Scotland, ‘value for money’ decisions for genetic testing are often made based on cost effectiveness, with a focus on diagnostic yield, as well as budget impact considerations [39]. Additionally, qualitative pilot work in a small sample of SGP and DDD study participants [7] indicated that quality-adjusted life years (QALYs), the standard health economic utility measure, may not be sensitive to changes in patients’ and families’ utility. However, recent economic evaluations of genome-wide sequencing have employed cost-utility analysis (CUA), using QALYs as the outcome measure [37, 40, 41]. These studies have assessed the value for money offered by genome-wide sequencing in terms of long-term costs and consequences such as changes in clinical management, avoided tests and clinic visits, and increases in life expectancy. The focus on diagnostic yield using a CEA framework does not account for these long-term outcomes.

Whilst the chance of diagnosis is clearly fundamental, focusing on diagnostic yield alone also fails to account for the broader value of genome-wide sequencing to patients and families with rare conditions. Previous research has found that service users value a wide range of clinical, informational, process and psychological factors associated with GS, beyond the chance of diagnosis [7, 42–44]. This includes changes in clinical management, access to support and services, information for family planning, waiting times for genetic testing results, relief, peace of mind and closure. Within the economic evaluation literature, recent studies have attempted to value these broader factors using patient preference data and cost–benefit analysis (CBA) modelling [36, 45]. In future research, we will use cost–benefit analysis to evaluate the broader utility of genome-wide sequencing to patients and families with undiagnosed rare conditions.

## Conclusion

The Scottish NHS is currently considering which genetic and genomic testing services to provide for the diagnosis of rare developmental disorders. This study found that offering trio ES as a second-line test (after CMA, but replacing gene panels) is a cost-saving option for the Scottish NHS, compared to the diagnostic odyssey of genetic testing. For strategies involving GS, despite the small increase in diagnostic yield, costs increased significantly. This suggests that, at present, WTP per additional diagnosis would need to be £48,000–£83,000 (depending on trio GS costs) to justify the additional cost of GS, compared to ES. Whilst several areas for future research have been identified, our results remain useful in planning a Scottish genetic and genomic testing strategy for undiagnosed developmental disorders over the short- to medium-term.

## Supplementary Information

Below is the link to the electronic supplementary material.

Supplementary file1 (DOCX 255 KB)

## Data Availability

The authors of this manuscript are willing to share all de-identified summary datasets and protocols used in the development of the cost-effectiveness analysis model upon reasonable request. However, datasets which contain identifiable patient-level data cannot be shared. Further information on the model inputs and economic evaluation methods can also be found in the online supplementary material.
