# Supplementary material for: Should Scotland provide genome-wide sequencing for the diagnosis of rare developmental disorders? A cost-effectiveness analysis
Source: Eur J Health Econ. 2024 Sep 9;26(3):503–12. doi: 10.1007/s10198-024-01717-8 (PMC11937054; doi:10.1007/s10198-024-01717-8)
Supplement: Supplementary file 1 — Supplementary file1 (DOCX 255 KB) [file 10198_2024_1717_MOESM1_ESM.docx]

**Online Supplementary Material**

**Should Scotland Provide Genome-Wide Sequencing for the Diagnosis of Rare Developmental Disorders?** A Cost-Effectiveness Analysis

European Journal of Health Economics

Michael Abbott^1*^, Mandy Ryan^1^, Rodolfo Hernández^1^, Lynda McKenzie^1^, Sebastian Heidenreich^2^, Lynne Hocking^3,4^, Caroline Clark^4^, Morad Ansari^5^, David Moore^5^, Anne Lampe^5^, Ruth McGowan^6^, Jonathan Berg^7^ and Zosia Miedzybrodzka^3,4^

^1^ Health Economics Research Unit, University of Aberdeen, Aberdeen, UK

^2^ Evidera Inc., London, UK

^3^ Department of Medical Genetics, University of Aberdeen, Aberdeen, UK

^4^ NHS Grampian Regional Genetics Service, Aberdeen Royal Infirmary, Aberdeen, UK

^5^ South East Scotland Genetic Service, NHS Lothian, Edinburgh, UK

^6^ West of Scotland Centre for Genomic Medicine, QEUH, Glasgow, UK

^7^ NHS Tayside Genetics Service, Dundee, United Kingdom

*Corresponding Author: Michael Abbott, Research Fellow, Health Economics Research Unit, University of Aberdeen. Email: [Michael.abbott@abdn.ac.uk](mailto:Michael.abbott@abdn.ac.uk) Tel: +44 (0) 1224 437 199

Cost-Effectiveness Modelling

Costing Standard Genetic Testing

The cost of the genetic testing histories of Scottish Genomes Partnership (SGP) and Deciphering Developmental Disorders (DDD) study participants was estimated. The cost of each genetic test was estimated using the GENU; a workload unit-based method developed by the UK Genetic Testing Network^1^ in the assessment of clinical genetic activities in UK laboratories linked to the agreed Scottish Genetics Laboratories cost/workload unit (WLU). Each genetic test was placed into one of eight costing bands, which reflects the complexity of the test in terms of the number of genes/gene fragments involved, as well as staff and laboratory inputs and overheads. The cost of each genetic test therefore reflects the total laboratory workload associated with delivering the genetic test.

The cost of genetics clinic visits was also estimated for SGP and DDD study participants. For appointments with a genetics consultant, a cost of £396 per appointment was used.^2^ For appointments with a genetic counsellor or nurse, the Personal Social Services Research Unit (PSSRU) cost per minute was used.^3^ The length of a genetic counsellor or nurse appointment was estimated based on the average length of an appointment in the SGP study. Although many appointments were conducted online throughout the Covid-19 pandemic, it was assumed that standard clinical practice going forward would involve face-to-face appointments.

The standard genetic testing costing focused on probands’ last five years of genetic testing and clinic visits, prior to their enrolment in the SGP or DDD study. The focus on more recent testing aimed to exclude any irrelevant and/or outdated tests which may not reflect future clinical practice in Scotland. For example, many single gene tests may have been replaced by larger gene panels. Including the cost of older single gene tests would therefore fail to reflect current Scottish clinical practice, and may lead to double counting of health care resource use.

First-line genetic testing typically included a combination of Chromosomal Microarray (CMA) and Fragile X testing. Developmental disorder probands had a mean first-line genetic testing cost of £386 (95% CI £358 - £414).

For second-line standard genetic testing, a series of gene tests and gene panel tests made up the bulk of the diagnostic odyssey. On average, developmental disorder patients had 3.7 tests and 3.3 genetics clinic visit over the course of their diagnostic trajectory. The mean cost of second-line genetic testing was £899 (95% CI £845 - £953), while the mean cost of genetics clinic visits was £1,376 (95% CI £1,313 - £1,440). This gave a total second-line genetic testing cost of £2,275 (95% CI £1,836 - £2,948).

Costing Trio Genome Sequencing (GS)

The cost of delivering trio GS was estimated using micro-costing^4^ (as no GENU category exists for this test). Two separate costing scenarios were generated following the conclusion of the SGP study. Firstly, the cost of trio GS was estimated according to the protocol used for the SGP’s involvement in the UK 100,000 Genomes Project. A previous costing study^5^ estimated the cost of trio-based GS using this protocol. However, the SGP study involved significant costs for infrastructure setup, participant screening and recruitment. These costs would not apply if GS were delivered in Scottish clinical practice. This protocol was set up 7 years ago and therefore the GS pipeline was therefore updated to attempt to reflect changes in the cost of delivering GS since the conclusion of the SGP study, and to reflect the cost of delivering GS in clinical practice rather than in a research context. An overview of the GS pipeline using a similar protocol to the SGP study is presented in Figure S1.

Figure S1: GS Using a Similar Pipeline to the SGP Study


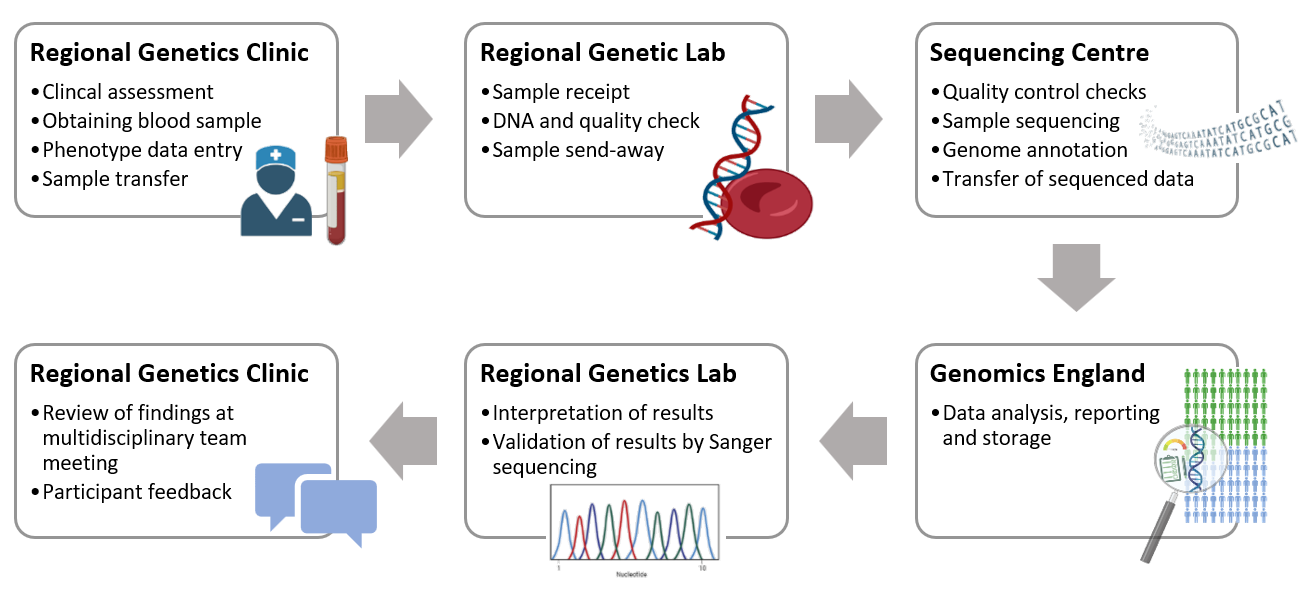


Table S1 details the costs involved at each stage of the GS process, using a similar protocol to the SGP study.

Table S1: Cost of Trio GS as Delivered in the SGP Study

| **Cost Component** | **Estimated Cost per Trio** | **% of Total GS Cost** |
| --- | --- | --- |
| Genetics Clinic Costs  (Phenotyping, MDT meeting, Participant Feedback) | £296.96 | 5.3% |
| Genetics Lab Costs  (Sample reception, DNA Extraction, Variant Interpretation, Validation and Reporting) | £964.21 | 17.3% |
| Sequencing Provider | £3,555 | 63.8% |
| Bioinformatic Analysis | £525 | 9.4% |
| Data Storage | £235 | 4.2% |
| **Total Cost per Trio** | £5,576.16 | |

A second GS costing scenario was considered, which involved outsourcing the sequencing stage of GS to Genomics England. This would entail conducting the sequencing, data analysis and storage of genomic data at Genomics England. All other stages of the GS process (e.g. DNA sample taking and extraction, library preparation, variant interpretation and patient feedback) would still occur at Scotland’s regional genetics centres. As with the first GS costing scenario, research study costs such as participant recruitment were excluded. In this scenario, it was estimated that GS could be provided at a cost of £2,520 per trio (based on discussions with Genomics England around GS service delivery). This cost includes sequencing, analysis and storage of genomic data. The reduction in the cost of sequencing gives a total GS cost per trio of £3,781.16. This was considered the lower bound of the trio GS cost in the economic modelling. Conducting the sequencing at Genomics England reduces its contribution to the overall GS cost to 50%. This suggests that conducting sequencing at Genomics England is a cheaper alternative, assuming Genomics England are able to provide the service for NHS Scotland at the price assumed here.

Costing Trio Exome Sequencing (ES)

Similar to the GS costing, the cost of delivering trio-based ES was estimated using micro-costing. The micro-costing exercise was conducted at South East Scotland Genetic Service, where trio ES is currently offered for severe developmental disorders as an NHS diagnostic service. Detailed inputs of the staff time, consumables and equipment used at each stage of the ES pipeline were recorded to estimate the cost of trio-based ES. The trio ES pipeline involves patient recruitment at each of the four clinical genetics centres in Scotland, sample taking, and DNA extraction at local genetics laboratories. This is followed by library preparation; whole exome capture and quality control at South East Scotland Genetic Service. Libraries in batches of 96 (32 trios) are transferred to Edinburgh Genomics at the University of Edinburgh for paired-end sequencing on an S2 flow cell on the NovaSeq 6000, with a turnaround time of 2-5 days. Following sequencing, the sequence data are quality controlled and released through an encrypted transfer server to a dedicated bioinformatics team at the Institute of Genetics and Cancer (IGC) who download the data to Edinburgh Parallel Computing Centre (EPCC), where variant annotation and filtering identifies potentially causative genotypes. Variants are then transferred to Clinical Scientists at South East Scotland Genetic Service for classification using DECIPHER (https://www.deciphergenomics.org/). All significant variants are confirmed by Sanger sequencing. If required, a multi-disciplinary team meeting is also conducted. Finally, a diagnostic report is issued to the referring clinician which is then discussed with the patient and their family. *Table S2* indicates the cost of each component of the trio ES pipeline, as well as the contribution of each component to the overall cost:

Table S2: Cost of Trio ES at South East Scotland Genetic Service

| **Cost Component** | **Estimated Cost Per Trio** | **% Of total ES cost** |
| --- | --- | --- |
| Sample taking and DNA Extraction | £65.24 | 5.7% |
| DNA Sample Library Preparation | £248.35 | 21.5% |
| Sequencing | £546.88 | 47.4% |
| Bioinformatic analysis | £83.99 | 7.3% |
| Variant Classification | £57.71 | 5.0% |
| Validation and Reporting incl. MDT | £91.44 | 7.9% |
| Feedback to Patients | £60.13 | 5.2% |
| **Total Cost per Trio** | **£1,153.74** | - |

Overall, it was estimated that trio ES costs £1,153.74 per trio. Similar to the trio GS costs, a large proportion (47.4%) of the overall ES cost was accrued at the sequencing stage, with a further 7.3% and 5.0% for bioinformatics and variant classification, respectively.

Estimating Diagnostic Yield of Alternative Strategies

Diagnostic yield (the proportion of cases receiving a positive genetic diagnosis) was used as the clinical effectiveness measure in the CEA model. A variety of data sources were used to identify diagnostic yield statistics for clinical effectiveness inputs. This included data from the SGP and DDD study reports, review of the clinical and economic literature evaluating the (cost) effectiveness of genome-wide sequencing, and expert genetic opinion. Table S3 summarises the diagnostic yield for each genetic and genomic test, as well as the data sources and probability distributions used in the probabilistic sensitivity analysis.

Table S3: Summary of CEA Diagnostic Yield Inputs, Distributions and Data Sources

| Test and Timing in Care Pathway | Diagnostic Yield  (95% CI) | Distribution | Data Source |
| --- | --- | --- | --- |
| First-line array/Fragile X | 0.10  (0.09 – 0.12) | Beta  (154, 1,382) | Ontario Health (2020) |
| Second-line single gene tests and panels | 0.21  (0.14 – 0.29) | Beta  (24, 89) | Ontario Health (2020) |
| Last-resort GS | 0.23  (0.14 – 0.32) | Beta  (25, 66) | SGP study and 100,000 Genomes Project diagnostic yield reports |
| Last-resort ES | 0.21  (0.13 – 0.29) | Beta  (22, 68) | DDD Study diagnostic yield report (Firth and Wright, 2015) |
| Second-line GS | 0.40*  (0.33 – 0.47) | Beta  (34, 50) | *No data – assumed to be between 1^st^- and 2^nd^-line GS |
| Second-line ES | 0.37  (0.27 – 0.49) | Beta  (33, 56) | Ontario Health (2020) + NHS Scotland trio ES DDG2P yield |
| First-line GS | 0.46  (0.36 – 0.57) | Beta  (31, 36) | Ontario Health (2020) |

For standard genetic testing, including chromosomal microarray, Fragile X, single gene tests and gene panel testing, systematic review data^7^ was used to estimate diagnostic yield. It was estimated that first-line array and Fragile X testing has a diagnostic yield of 10% (95% CI 0.09 – 0.12), while second-line single gene tests and gene panels provide a further 21% yield (95% CI 0.14 – 0.29).

The SGP and DDD research studies offered insight into the diagnostic yield of trio GS and ES *as a last-resort* test, when all prior testing had failed to reach a diagnosis. This is a result of the eligibility criteria of the SGP and DDD studies, which required that participants had exhaustive standard genetic testing prior to enrolment in the study, where they received GS or ES. The SGP study estimated that last-resort GS gave a diagnostic yield of 23% (95% CI 0.14 – 0.32). This is *in addition to* the yield of prior array, fragile X, single gene and gene panel testing. The DDD study estimated that last-resort ES gave a diagnostic yield of 21% (95% CI 0.13 – 0.29). Again, this yield is in addition to any prior standard genetic testing.

Trio ES is currently offered in NHS Scotland for the diagnosis of severe developmental disorders. This provided insight into the diagnostic yield of 2^nd^-line ES, after 1^st^-line array and fragile X testing, but replacing single gene tests and gene panels. Analysis of the Developmental Disorder Genotype-to-Phenotype (DDG2P) gene panel (PMID 31147538) in a total of 776 trios gave a diagnostic yield of 37% (95% CI 0.27 – 0.49) for ES as a 2^nd^-line test.

No Scottish data was identified for the diagnostic yield of first-line GS. As a result, systematic review data^7^ was used to estimate the diagnostic yield of GS as a first-line test. It was estimated that 1^st^-line GS provides a diagnosis in 46% of cases (95% CI 0.36 – 0.57). Additionally, no Scottish data and no systematic review data was identified for the diagnostic of 2^nd^-line GS. As a result, an assumption was made that the diagnostic yield of 2^nd^-line GS is between the yield of 1^st^-line GS and last-resort GS. The diagnostic yield of 2^nd^-line GS was assumed to be 40% (95% CI 0.33 – 0.47).

1^st^-line trio ES was not modelled as an alternative genomic testing strategy as, at the time of model conception and development, expert clinical opinion indicated that a first-line chromosomal microarray and/or fragile X test would always occur prior to ES. This was due to the fact that ES was unable to detect copy-number variations, and array and fragile X offer a relatively inexpensive means of detecting these variations.

Sensitivity Analysis: Lower WGS Costs (£3,781 per trio)

*Figure S2* presents the efficiency frontier for the deterministic sensitivity analysis using a lower cost of £3,781 per trio, with sequencing, bioinformatic analysis and data storage outsourced to Genomics England.

**Figure S3: Lower GS Cost Efficiency Frontier: GS Cost = £3,781 per trio**

Given that the ES cost remained the same in both scenarios, 2^nd^-line ES remained a cost-saving option compared to standard genetic testing. 2^nd^-line trio ES offered cost savings of £7,388 per additional diagnosis. Compared to 2^nd^-line ES, 1^st^-line GS had an incremental cost of £61,000 per additional diagnosis. Last-resort ES was dominated by 1^st^-line GS, and 2^nd^-line GS was dominated by last-resort ES. Compared to 1^st^-line GS, last-resort GS had an incremental cost of £1,152,000 per additional diagnosis, with a £1,152 increase in cost and 0.1% increase in diagnostic yield.

Probabilistic Sensitivity Analysis

*Figure S3* illustrates the cost-effectiveness acceptability curve (CEAC) for the base case analysis, where GS costs £5,576 per trio. The CEAC illustrates the probability that each strategy is cost effective, for WTP values ranging from £0 to £100,000 per additional diagnosis. 2^nd^-line ES was a dominant strategy, with a lower cost and higher diagnostic yield than standard care. Consequently, 2^nd^-line ES was most likely to be cost effective at all WTP values up to £83,000 per additional diagnosis. As WTP per additional diagnosis increases, the probability of alternative strategies being cost effective increases. Beyond £83,000 per additional diagnosis, 1^st^-line GS becomes most likely to be cost effective. Further, as WTP increases, 2^nd^-line GS, last-resort ES and last-resort GS become more (but not most) likely to be cost effective, as WTP increases.


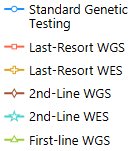

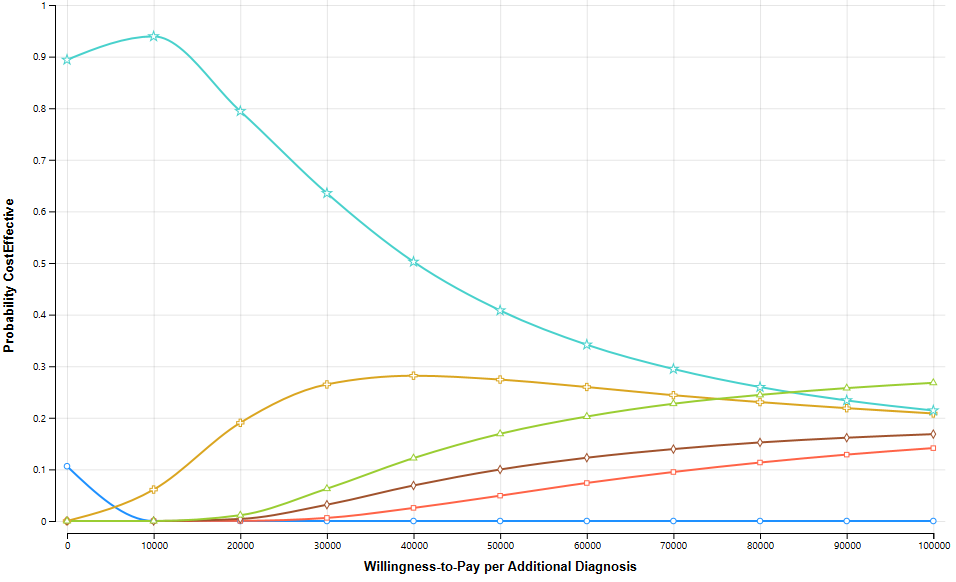
**Figure S3:** Cost-Effectiveness Acceptability Curve (Base Case Analysis)

In the lower GS cost scenario (When trio GS costs £3,781), first-line GS becomes more likely to be cost-effective at WTP values greater than £48,000 per additional diagnosis. At the implied WTP value of £8,613 per additional diagnosis, 2^nd^-line ES had a 73% probability of being cost effective. As the GS cost decreases, WTP per additional diagnosis does not need to be as high before GS strategies become cost effective.

**References**

1. UK Genetic Testing Network (2016). Development of GenUs
2. Public Health Scotland. (2021). Scottish Health Service Costs. Available Online at: [https://publichealthscotland.scot/publications/scottish-health-service-costs/scottish-health-service-costs-high-level-costs-summary-2020-to-2021](https://publichealthscotland.scot/publications/scottish-health-service-costs/scottish-health-service-costs-high-level-costs-summary-2020-to-2021/)
3. PSSRU. (2021). Unit Costs of Health and Social Care 2021. Available Online at: [https://www.pssru.ac.uk/project-pages/unit-costs/unit-costs-of-health-and-social-care-2021](https://www.pssru.ac.uk/project-pages/unit-costs/unit-costs-of-health-and-social-care-2021/)
4. Frick (2009). Micro-Costing Quantity Data Collection Methods. *Medical Care,* 47, 76 – 81.
5. Abbott et al. (2021). Continuing the Sequence? Towards an economic evaluation of whole genome sequencing for the diagnosis of rare diseases in Scotland. Journal of Community Genetics, 13, 487 – 501. <https://doi.org/10.1007/s12687-021-00541-4>
6. Ontario Health (Quality). (2020). Genome-wide sequencing for unexplained developmental disabilities or multiple congenital anomalies: a health technology assessment. *Ont Health Technol Assess Series,* 1–178.
